# Confronting Sulfur Electrode Passivation and Li Metal Electrode Degradation in Lithium‐Sulfur Batteries Using Thiocyanate Anion

**DOI:** 10.1002/advs.202301006

**Published:** 2023-03-21

**Authors:** Jinkwan Jung, Hyunwon Chu, Ilju Kim, Dong Hyun Lee, Gisu Doo, Hyeokjin Kwon, Wonhee Jo, Sejin Kim, Hyenah Cho, Hee‐Tak Kim

**Affiliations:** ^1^ Department of Chemical and Biomolecular Engineering KAIST 291 Daehak‐ro, Yuseong‐gu Daejeon 34141 Republic of Korea; ^2^ Advanced Battery Center KAIST Institute for the NanoCentury KAIST 291 Daehak‐ro, Yuseong‐gu Daejeon 34141 Republic of Korea

**Keywords:** acceptor number, Gutmann donor number, Li^+^ solvation shell, Li_3_N‐rich SEI layer, lithium sulfur batteries, thiocyanate anions

## Abstract

Salt anions with a high donor number (DN) enable high sulfur utilization in lithium‐sulfur (Li‐S) batteries by inducing three‐dimensional (3D) Li_2_S growth. However, their insufficient compatibility with Li metal electrodes limits their cycling stability. Herein, a new class of salt anion, thiocyanate (SCN^−^), is presented, which features a Janus character of electron donor and acceptor. Due to a strong Li^+^ coordination by SCN^−^ and the direct interaction of SCN^−^ with polysulfide anions, the LiSCN electrolyte has a remarkably high lithium polysulfide solubility. This electrolyte induces 3D Li_2_S formation and ameliorates cathode passivation, even more than Br^−^, a typical high DN anion. Moreover, SCN^−^ forms a Li_3_N‐enriched stable SEI layer at the surface of the Li metal electrode, enhancing cycling stability. A Li‐S battery with the LiSCN electrolyte shows high current density operation (2.54 mA cm⁻^2^) with high discharge capacity (1133 mAh g⁻^1^) and prolonged cycle life (100 cycles). This work demonstrates that the cathode and anode performance in a Li‐S battery can be simply and concurrently enhanced by the single salt anion.

## Graphical abstract

This study investigates the potential of lithium thiocyanate (LiSCN) salt to improve both electrodes by enhancing sulfur utilization and Li metal stability. Based on the high donor number and acceptor number SCN^−^ anion, the intensified 3D growth of lithium sulfide is induced on the sulfur electrode and the robust Li_3_N solid electrolyte interphase layer is constructed on the Li electrode.

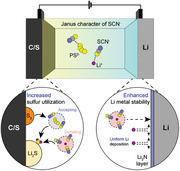

## 1 Introduction

Lithium‐sulfur (Li‐S) batteries have been intensively researched as promising post lithium‐ion batteries due to their high theoretical energy density.^[^ ^1^, ^2^, ^3^ ^]^ Despite past efforts, cathode passivation by insulating Li_2_S, which practically limits sulfur utilization, remains a challenge. A film‐like Li_2_S growth on carbon surface prevents the complete conversion of polysulfides (PSs), which becomes more severe at a low electrolyte to sulfur (E/S) ratio and high current density or areal capacity.^[^ ^4^, ^5^, ^6^, ^7^, ^8^ ^]^ Previous works on the effect of solvent donor number (DN) on Li_2_S morphology provide important insight on the possibility of electrolyte‐driven control of the Li_2_S growth mechanism.^[^ ^9^, ^10^, ^11^, ^12^, ^13^, ^14^ ^]^ Pan et al. suggested an increased solubility of PS species with a high DN solvent, DMSO.^[^ ^15^ ^]^ Li et al. reported retarded Li_2_S nucleation due to the stabilization of long chain PSs in high DN solvents (DMSO, DMA, MeIm).^[^ ^9^ ^]^ Despite an encouraging high sulfur utilization at the first cycle, high DN solvents are highly corrosive to Li metal electrodes, limiting the positive effect only to a few initial cycles. Recently, Elabd et.al reported a novel high DN solvent, 3‐fluoropyridine, which has high PS solubility and compatibility with a Li metal electrode, demonstrating the potential of a high DN electrolyte in designing a practical Li‐S battery.^[^ ^16^ ^]^

High DN salt anions provide another means to promote solution‐mediated 3D Li_2_S growth.^[^ ^12^ ^]^ Br^−^, NO_3_^−^, and CF_3_SO_3_^−^ anions, the DNs of which are 33.7, 22.2, and 16.9 kcal mol⁻^1^, respectively, lead to 3D Li_2_S growth and consequent high sulfur utilization in contrast to 2D growth with the conventional TFSI^−^ anion (DN: 5.4 kcal mol⁻^1^). In particular, the LiBr‐based electrolyte exhibited 92% sulfur utilization for 80 cycles, shedding light on achieving high sulfur utilization. However, the advantages of high DN salt anions are screened by insufficient Li metal cycling stability and low Li^+^ conductivity. LiBr‐based electrolytes typically illustrate this issue; Br^−^ has the most pronounced effect on suppressing electrode passivation, but it is less compatible with a Li metal electrode compared with lower DN salt anions.

Electron accepting ability, which is represented by the acceptor number (AN), can provide another solvation chemistry to amend the Li_2_S growth mode. In Li‐oxygen batteries, 3D Li_2_O_2_ growth is induced by a high AN solvent such as H_2_O.^[^ ^17^, ^18^ ^]^ This directly solvates O_2_^−^, increasing the solubility of Li_2_O_2_. In a similar way, a high AN molecule may solvate S^2−^ or various PS anions, inducing 3D Li_2_S growth. However, high AN molecules are prone to decomposition at the Li metal electrode due to their electrophilicity, which imposes a limitation for their use in Li‐S batteries.

Herein, we present a new class of lithium salt anion, thiocyanate (SCN^−^), which possesses an electron donating and accepting property, for Li‐S batteries. The electron deficient carbon atom and electron abundant nitrogen and sulfur atom provide high AN and high DN function, respectively. The electrolyte with an SCN^−^ anion can retard electrode passivation and exhibits a sulfur utilization even greater than that with the Br^−^ anion. More importantly, the LiSCN electrolyte is highly compatible with Li metal electrodes, enhancing their cycling stability in lean electrolyte and high current density conditions. We suggest the underlying mechanisms for positive effects based on the solvation chemistry derived from the interaction of SCN^−^ with Li^+^ and PS anions. A Li‐S battery with our proposed solvation chemistry demonstrates suppressed electrode passivation, high current density operation, and excellent Li metal stability, presenting a novel route to advance Li‐S batteries.

## 2 Results and Discussion

### 2.1 Solvation Chemistry Based on Janus Character of SCN^−^ Anion

**Figure 1:**
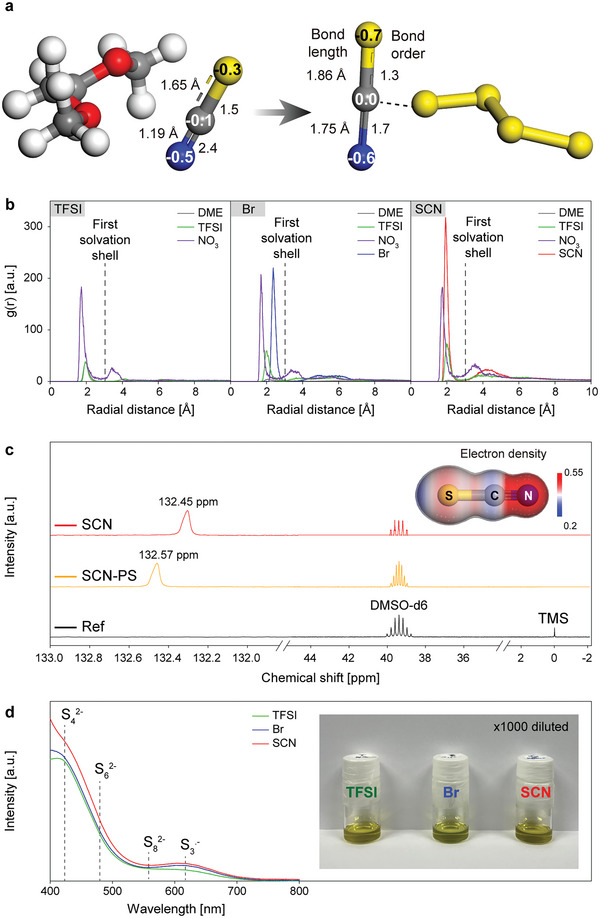
Solvation chemistry of Li^+^ and PSs with SCN^−^ a) Changes in partial charge, bond length, and bond order of the SCN anion calculated by the Mulliken charge analysis method of DFT simulations by the interaction with DME or S_4_^2−^. b) Radial distribution function of Li^+^ for solvents and anions in the LiTFSI, LiBr, and LiSCN electrolytes. Dotted lines indicate the criterion of the first solvation shell of Li^+^. c) ^13^C NMR spectra of the LiSCN electrolyte with and without 1.6 m Li_2_S_4_. d) Optical images and UV–vis spectra of the diluted catholyte solution for the LiTFSI, LiBr, and LiSCN electrolytes.

The Janus character of SCN^−^ is typically represented by its electronic structure. As indicated by DFT calculation (Figure S2, Supporting Information), electro‐negative N and S atoms draw electrons from carbon atoms, resulting in a localized electron distribution around the N and S atoms.^[^ ^19^, ^20^ ^]^ The charge distributions on N, S, and C are ‐0.51e, ‐0.72e, and 0.23e, respectively (**Figure** **1**a). Therefore, the electron‐deficient C atom can function as an electron accepting site and the S and N atom can function as an electron donating site. Due to the electron donating atoms, SCN^−^ has 2.3 times higher DN (25.6 kcal mol⁻^1^) than TFSI^−^ (11.2 kcal mol⁻^1^); however, it is lower than that of Br^−^ (33.7 kcal mol⁻^1^).^[^ ^21^ ^]^

The electron donating ability of SCN^−^ can be demonstrated by the Li^+^ solvation structure because the first Li^+^ solvation shell is mainly composed of high donating solvents or salt anions. For each LiTFSI, LiBr, and LiSCN electrolyte, a molecular dynamics (MD) simulation was conducted and their Li^+^ solvation structures are compared in Figure 1b. As presented in the radial distribution functions (Figure 1b), DME solvent is the major component of the solvation shell for the LiTFSI electrolyte, whereas it is not for the LiBr and LiSCN electrolyte, as indicated by the coordination numbers of DME (1.73, 0.35, and 0.21 for the LiTFSI, LiBr, and LiSCN electrolyte, respectively) (Figure S3, Supporting Information). The coordination numbers of SCN^−^ (1.75) and Br^−^ (1.88) are much higher than that of TFSI^−^ (0.59), indicating that SCN^−^ and Br^−^ molecules readily participate in Li^+^ solvation. The MD simulation results clearly show the high DN characteristic of SCN^−^.

However, being distinguished from other high DN salt anions, SCN^−^ directly interacts with PS anions due to its electron accepting property. The interaction between PS anions and SCN^−^ was investigated using ^13^C NMR spectra of the LiSCN electrolytes with and without 1.6 m Li_2_S_4_. An environmental change of SCN^−^ caused by the PS‐SCN^−^ interaction can lead to a change in the C chemical shift of SCN^−^.^[^ ^13^ ^]^ As shown in Figure 1c, the signal from SCN^−^ shifted downfield when adding Li_2_S_4_, indicating a de‐shielding effect by the PS anions and a change of the local environment of SCN^−^ by the PS anions. We expect the carbon of SCN^−^ interacts with the oxygen of DME solvent in the absence of the PS anions, and the replacement of DMEs with PS anions resulting in the de‐shielding effect. Due to the direct interaction of S_4_^2−^ with SCN^−^ in replacement of DME, the charge density of the carbon of SCN^−^ is decreased which is supported by the DFT calculation (Figure 1a). The electron acceptor property of SCN^−^ was further demonstrated using Raman spectroscopy analysis. The Raman shift of the antisymmetric CN stretching (2071 cm⁻^1^) for the LiSCN electrolyte was decreased to a lower wavenumber (2067 cm⁻^1^) with the addition of 1.6 m Li_2_S_4_, as shown in Figure S4 (Supporting Information).^[^ ^22^ ^]^ Strong coordination of PS anion to C atom of SCN^−^ elongates the CN bond and decreases the bond order, resulting in the red shift of the CN stretching mode, thereby weakening the CN bond.

In the LiPS‐containing electrolyte, various PSs with different orders coexist and their relative portions are varied depending on the electrolyte. To prepare PS‐saturated electrolytes, we added 1.6 m Li_2_S_4_ to the LiTFSI, LiBr, LiSCN electrolytes and diluted the supernatant of each catholyte by 1000‐fold for UV–vis analysis. The UV–vis spectra of the supernatant inform the PS solubility and preferred PS species of the corresponding electrolyte. As shown in Figure 1d, the diluted supernatants of the PS‐saturated LiTFSI, LiBr, and LiSCN catholytes (colored yellow, green, and dark yellow, respectively) indicate different distributions of the PS species for the electrolytes. The UV–vis spectra for the LiBr and LiSCN electrolytes exhibited a strong absorption peak at 617 nm corresponding to S_3_^.−^, which is typical of high DN electrolytes.^[^ ^23^, ^24^ ^]^ In the case of the LiTFSI electrolyte, S_4_^2−^ was dominant but S_3_^.−^ was not the main PS species due to its low DN in accordance with previous research.^[^ ^24^, ^25^, ^26^ ^]^ It is remarkable that the LiSCN electrolyte showed the highest absorbance in the whole range of 400–800 nm, indicating the highest solubility, regardless of PS type. The high absorbances of S_8_^2−^, S_6_^2−^, and S_3_^.−^ for the LiBr and LiSCN electrolytes can be explained in terms of DN.^[^ ^27^ ^]^ The high DN anions strongly associate with Li^+^, which leads to the dissociation of various LiPSs.

On the other hand, the comparison of the absorbances from S_4_^2−^ and S_3_^.−^ provides evidence for the stabilization of hard basic S_4_^2−^ by electron‐donating SCN^−^. The absorbance ratio of S_4_^2−^ to S_3_^.−^ is 8.0 for the LiBr electrolyte and 8.5 for the LiSCN electrolyte, which indicates that S_4_^2−^ is more stabilized in the LiSCN electrolyte due to the electron sharing between S_4_^2−^ and the carbon atom of SCN^−^. The UV–vis analysis suggests that SCN^−^ can increase the solubility of LiPSs owing to its Janus character.

### 2.2 Suppression of Sulfur Electrode Passivation by SCN^−^ Anion

**Figure 2:**
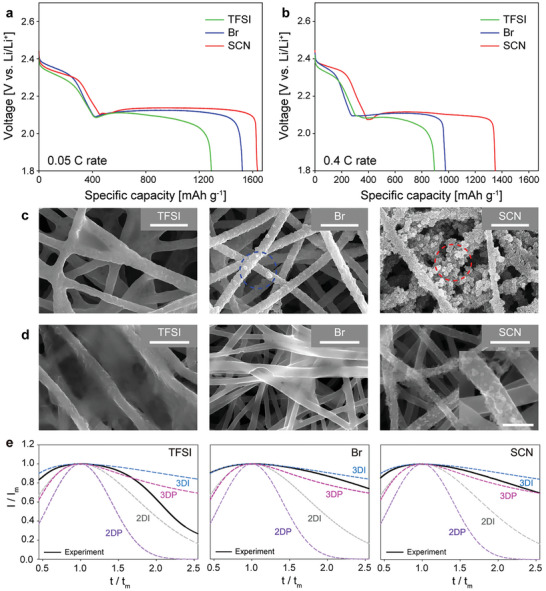
Discharge behavior, Li_2_S deposition morphologies, and Li_2_S growth modes for the LiTFSI, LiBr, and LiSCN electrolytes. Discharge profiles for the Li‐S cells with the LiTFSI, LiBr, and LiSCN electrolytes at the a) 0.05 C rate and b) 0.4 C rate. Li_2_S deposition morphologies in the cathode for the three electrolytes after discharge at c) 0.05 C and d) 0.4 C. e) Current density‐time transient curves for the chronoamperometry results obtained with the three electrolytes. Theoretical responses for 2D/3D and instantaneous/progressive nucleation are indicated. (Scale bars indicate 2 µm and scale bar for the inset figure of Figure 2d indicates 500 nm).

**Figure** **2**a compares the first discharge profile at 0.05 C among the LiSCN, LiTFSI, and LiBr electrolytes. Remarkably, the LiSCN electrolyte showed 97.7% sulfur utilization (1636 mAh g⁻^1^) at 2 mg_s_ cm⁻^2^ loading at an E/S ratio of 20. This is much larger than that of the conventional LiTFSI electrolyte (1290 mAh g⁻^1^) and even larger than that of the LiBr electrolyte (1522 mAh g⁻^1^). At a higher discharge rate of 0.4 C, the salt anion tendency was consistently observed; the discharge capacity was 1349, 977, and 895 mAh g⁻^1^ for the LiSCN, LiBr, and LiTFSI electrolytes, respectively (Figure 2b).

After completing the first discharge at 0.05 C, the electrodes were taken out of the cells and subjected to SEM analysis (Figure 2c). The discharged electrode with the LiTFSI electrolyte was covered by a smooth film‐like Li_2_S deposit. In contrast, electrodes with the LiBr and LiSCN electrolytes exhibited nodular morphology of the Li_2_S deposit, indicating their 3‐D Li_2_S growth. Furthermore, the fine grain‐like Li_2_S morphology found for the LiSCN electrolyte indicates an intensified 3‐D Li_2_S growth. The ability of the LiSCN electrolyte to induce 3‐D Li_2_S morphology is more pronounced at the higher discharge rate of 0.4 C (Figure 2d, Figure S5, Supporting Information). As seen in Figure 2d, for the LiSCN electrolyte, porous electrode morphology was maintained due to the formation of particulate Li_2_S, which is contrasted by the passivated electrode surfaces for the LiTFSI and LiBr electrolytes. Despite the lower DN of SCN^−^ compared with that of Br^−^. SCN^−^, has a superior ability to retard passivation, which indicates that the electron accepting property of SCN^−^ contributes to the formation of 3‐D Li_2_S morphology.

To investigate the Li_2_S growth behavior, potentiostatic electrodeposition of Li_2_S was conducted using the Chronoamperometry (CA) technique. After a pre‐conditioning step to eliminate long‐chain PSs, the current responses were recorded by applying a step potential of 2.0 V (Figure S6, Supporting Information). The responses feature the appearance of the maximum current (I_max_) at a specific time (t_max_). Since the appearance of I_max_ originates from the overlapping of the nearby growing nuclei, t_max_ can be indicative of the relative speed of electrode passivation. The value for t_max_ was 735 s for the LiSCN, 645 s for the LiBr, and 228 s for the LiTFSI electrolyte, indicating slower passivation, respectively. For quantitative analysis, time and current were normalized by t_max_ and I_max_, respectively, and the plot of I/ I_max_ as a function of t/ t_max_ was compared for the three electrolytes. The response of the LiTFSI electrolyte lies in between the theoretical responses of 2D instantaneous growth and 3D progressive growth, whereas the responses of the LiSCN and LiBr electrolytes are in between the 3D progressive and 3D instantaneous growth (Figure 2e). By fitting an SH equation (Equation (S3), Supporting Information) to the CA data, the lateral growth rate of the Li_2_S nuclei (N_o_k_g_^2^) was estimated.^[^ ^28^, ^29^ ^]^ The lateral growth rate in the LiSCN electrolyte (3.81×10⁻^11^ s⁻^2^) was lower than that in the LiBr electrolyte (5.06×10⁻^11^ s⁻^2^) and in the LiTFSI electrolyte (1.44×10⁻^10^ s⁻^2^) (Table1, Supporting Information). The CA analysis is in line with the electrode morphologies, supporting the effective retardation of electrode passivation in the LiSCN electrolyte.

The Janus feature of the SCN^−^ anion exhibited distinguished Li_2_S and LiPS solubility behavior compared to the typical high DN (Br^−^) or low DN (TFSI^−^) electrolytes. It was reported that, for the high DN electrolyte, Li_2_S solubility is augmented by the common ion effect.^[^ ^12^ ^]^ The strong coordination of Br^−^ to Li^+^ decreases the concentration of free Li^+^, resulting in the shift of the equilibrium toward the dissociation of Li_2_S. For LiSCN electrolytes, Li_2_S dissolution can be achieved by not only the common ion effect but also the direct interaction between S^2−^ and electron accepting SCN^−^. As shown in Figure S7 (Supporting Information), LiTFSI, LiBr, and LiSCN electrolytes have a Li_2_S solubility of 4, 8, and 10 mm, respectively. The highest Li_2_S solubility of LiSCN electrolyte despite the lower DN than Br electrolyte indicates that the electron accepting C atom of SCN^−^ anion contributes to Li_2_S solubility.

The far retarded cathode passivation with LiSCN electrolyte can also be attributed to the intensified solution phase sulfur reduction with increased solubility of soft and hard basic PSs in LiSCN electrolyte. Until now, the theoretical capacity from the upper plateau in Li‐S battery has been accepted as 418 mAh g⁻^1^. This value assumes that S_4_^2−^ is the shortest PS dianion that can stably exist in solution during discharge. As seen in Figure 2a, the capacity from the higher voltage plateau is 446 mAh g⁻^1^ for LiSCN electrolyte, which is higher than the theoretical limit. It suggests that shorter polysulfides such as S_3_^2−^ can exist in solution phase. Since S_3_^2−^ is a hard base, it can be stabilized by the electron‐accepting C atom of SCN^−^. The chemical reactions of S_3_^2−^ (Equations (1), (2), (3)) suggest the formation of S^2−^ in the electrolyte phase, presenting a scenario of Li_2_S nucleation and growth in the electrolyte phase rather than on the electrode surface.^[^ ^30^, ^31^ ^]^

(1) $$6S_{3}^{2-}\to 4S_{4}^{2-}+2S^{2-}$$

(2) $$S_{3}^{2-}+S_{6}^{2-}\to S_{8}^{2-}+S^{2-}$$

(3) $$S_{3}^{2-}+S_{4}^{2-}\to S_{6}^{2-}+S^{2-}$$

Namely, the stabilization of shorter PSs by SCN^−^ provides additional chemical pathways for solution‐mediated Li_2_S deposition reaction and intensifies 3D Li_2_S morphology. The comparison of Raman spectra for 1.6 m Li_2_S_4_ containing LiTFSI, LiBr, and LiSCN catholytes (Figure S8, Supporting Information) reveals that the LiSCN electrolyte has a stronger peak intensity for both weak basic PSs (S_8_^2−^, S_7_^2−^, S_3_^.−^) and strong basic PSs (S_4_^2−^, S_3_^2−^), which is in good agreement with the UV–vis spectroscopy results obtained for the diluted LiPS catholytes (Figure 1d).^[^ ^32^, ^33^ ^]^ It confirms that various PSs including even hard basic S_3_^2−^ can stably exist in the SCN electrolyte.

### 2.3 Li Metal Stability with the LiSCN Electrolyte

**Figure 3:**
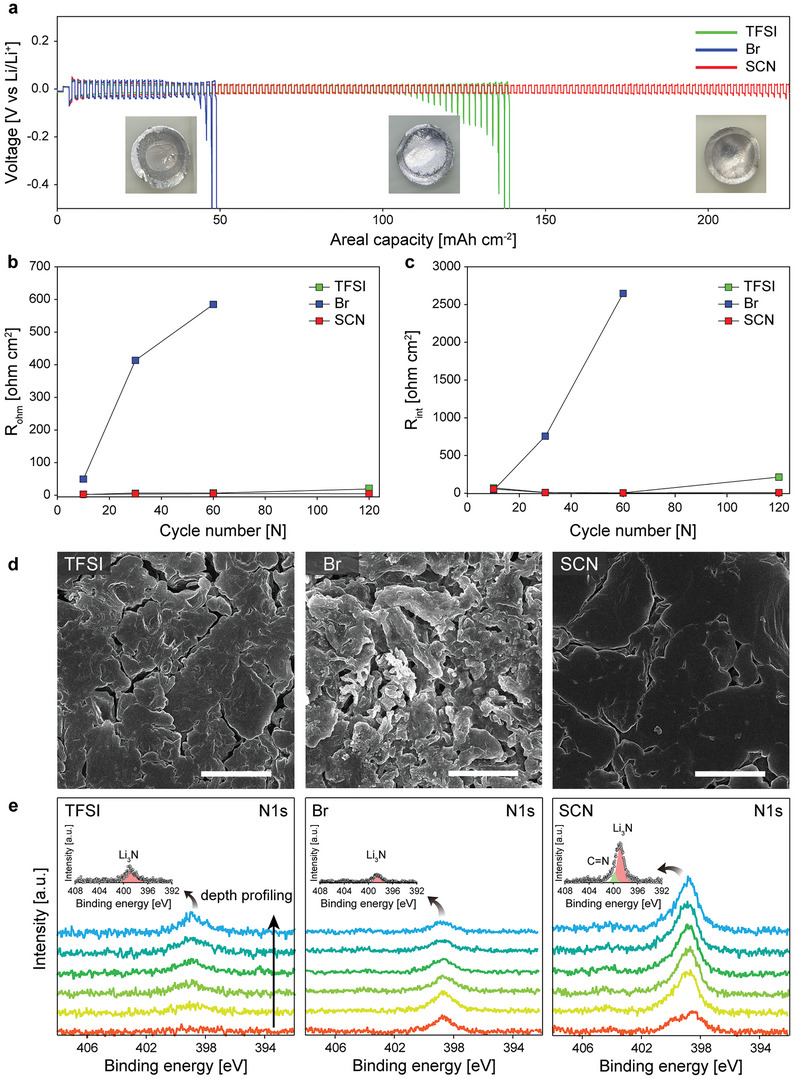
Li metal stability tests with electrochemical and post‐mortem analysis. a) Li/Li symmetric cell operation with the LiTFSI, LiBr, and LiSCN electrolytes in a 1 mA cm⁻^2^ / 1 mAh cm⁻^2^ condition for 225 mAh cm⁻^2^ (112 cycles). Inset figures present the optical images of the Li metal anodes taken out of the cycled cells after a 50 mAh cm⁻^2^ operation. b) Ohmic and c) Interfacial resistances of the Li/Li symmetric cells after 10, 30, 60, and 120 cycles. d) Surface images and e) N1s spectra of the Li metal surface after 20 cycles for the LiTFSI, LiBr, and LiSCN electrolytes. (Scale bars indicate 5 µm).

The development of a new electrolyte system directed at sulfur chemistry faces a challenge in stabilizing the Li metal electrode. Accordingly, the Li metal stability of the LiSCN electrolyte is of practical importance. To assess Li metal stability for the three electrolytes, the corresponding Li/Li symmetric cells were operated at 1 mA cm⁻^2^ / 1 mA h cm⁻^2^ with a 40 µm Li metal electrode. As shown in **Figure** **3**a, the LiBr electrolyte showed a sudden increase of polarization after 40 mAh cm⁻^2^, which is contrasted by the stable cycling of the LiTFSI electrolyte at more than 110 mAh cm⁻^2^. This result indicates that the LiBr electrolyte is not appropriate for use in practical Li‐S batteries despite its excellent ability to retard electrode passivation. Remarkably, the LiSCN electrolyte exhibited much higher cycling stability compared to the LiBr and LiTFSI electrolytes. This distinguished cycling stability clearly demonstrates the positive function of SCN^−^ in stabilizing the Li/electrolyte interface. This is significant because the use of a single component can address the two critical problems of cathode passivation and Li metal degradation.

To explore the performance fade mechanism of the Li metal electrode for the three electrolytes, EIS analysis was conducted during the cycling. Interfacial resistance (R_int_) and ohmic resistance (R_ohm_) were quantified by circuit model fitting (Figure S9, Supporting Information) and the results are compared in Figure 3b,c.^[^ ^34^ ^]^ The R_int_ reflects the accumulation of the resistive porous layer at the interface, and R_ohm_ indicates the electrolyte depletion caused by electrolyte decomposition. For the LiBr electrolyte, R_int_ and R_ohm_ increased notably with cycling, which indicates significant electrolyte decomposition and accumulation of the decomposed products on the Li metal surface. By sharp contrast, for the LiSCN electrolyte, R_int_ and R_ohm_ were stably maintained over the cycling, indicating excellent interfacial stability and suppressed electrolyte decomposition. In the initial cycles, the LiTFSI electrolyte also showed small and stable R_int_ and R_ohm_ values. However, at 120 cycles, the two resistances were increased, which is in line with the increased polarization (Figure 3a).

The SEM images of the Li electrolyte after 20 cycles showed distinguished Li deposit morphologies (Figure 3d). The LiTFSI electrolyte showed a granular Li deposit while the LiBr electrolyte showed finely fractured morphology. Remarkably, the LiSCN electrolyte exhibited relatively smooth and flat Li deposit morphology, which matches its excellent Li cycling stability. For the LiBr electrolyte, electrolyte decomposition is exacerbated with cycling due to the porous Li deposit morphology, leading to a resistive porous layer and consequent surge of polarization.

The chemical structure of SEI after 20 cycles was investigated by using X‐ray Photoelectron Spectroscopy (XPS). The N 1s spectra for the three electrolytes equivocally revealed a Li_3_N peak at 398.8 eV, which is known as a stable and ionic conductive SEI layer component (Figure 3e).^[^ ^35^, ^36^ ^]^ Since the base electrolyte used for the three electrolytes includes 0.2 m LiNO_3_, the three electrolytes commonly revealed the Li_3_N peak. For the LiSCN electrolyte, a C=N peak at 399.25 eV was examined and the intensity of the Li_3_N peak was much higher than the other electrolytes, which suggests the participation of SCN^−^ in the SEI formation.^[^ ^37^ ^]^ This result also presents a scenario in which the highly conductive Li_3_N‐enriched SEI layer is responsible for the enhanced cycling stability observed for the LiSCN electrolyte.

The C 1s spectra for the three electrolytes equivocally revealed C‐C, C‐O, and CO_3_^−^ peaks (Figure S10, Supporting Information). The O‐C=O peak derived from the solvent molecules^[^ ^38^ ^]^ was found for the LiBr and LiTFSI electrolytes, whereas it was unseen for the LiSCN electrolyte. The C‐H peak, which is indicative of solvent decomposition, was not detected for the LiSCN electrolyte. These observations indicate a suppressed solvent decomposition for the LiSCN electrolyte. The C‐S peak was found only for the SCN electrolyte, which supports the involvement of SCN^−^ in the SEI formation.^[^ ^39^ ^]^ The impedance, SEM, and XPS analysis collectively indicate that SCN^−^ actively contributes to SEI formation, and the resulting SEI layer enables uniform Li deposition, thus diminishing solvent decomposition.

The Li_3_N‐enriched SEI structures for the LiSCN electrolyte can be explained in terms of the Li^+^ solvation structure. The molecules participating in the solvation shell are more likely to populate at the surface of the Li metal electrode due to the continuous Li^+^ flux to the Li metal electrode during the Li deposition process. As the interfacial population is increased, the probability of reductive decomposition proportionally increases. In this aspect, for the LiTFSI electrolyte in which DME molecules mainly coordinate Li^+^, the reductive decomposition of DME is more significant. The largest XPS peaks from the organic components (Figure S10, Supporting Information) support this consideration. In the same way, the Br^−^ anion can highly populate at the interface for the LiBr electrolyte. However, Br^−^ does not form a stable and conductive SEI layer. For the LiSCN electrolyte, the populated SCN^−^ anions at the Li electrode surface result in a Li_3_N‐rich SEI layer, as demonstrated in the XPS analysis. The formation of a stable and ion‐conductive Li_3_N‐rich SEI layer leads to excellent cycling stability.

### 2.4 Electrochemical Performances of a Li‐S Battery with the LiSCN Electrolyte

**Figure 4:**
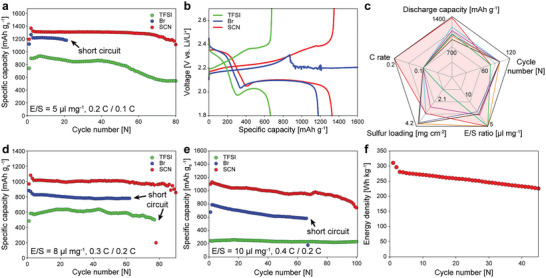
Li‐S full cell performance tests. a) Cycling performances of Li‐S cells at an E/S = 5 µl mg⁻^1^, 3 mg_s_ cm⁻^2^, and 0.2 C / 0.1 C rate for the LiTFSI, LiBr, and LiSCN electrolytes and b) the voltage profiles at the 21st cycle. c) Comparison of our Li‐S cell performances with recently reported other researches.^[^ ^41^, ^42^ ^]^ d) Cycling performances of the Li‐S cells at an E/S = 8 µl mg⁻^1^, 3 mg_s_ cm⁻^2^, and 0.3 C / 0.2 C rate for the three electrolytes. e) Cycling performances of the Li‐S cells at an E/S = 10 µl mg⁻^1^, 3.8 mg_s_ cm⁻^2^, and 0.4 C / 0.2 C rate for the three electrolytes. f) Cycling performances of a Li‐S pouch cell at an E/S = 4 µl mg⁻^1^, 3.17 mg_s_ cm⁻^2^, and 0.09 C / 0.09 C rate.

To demonstrate the advantages of the LiSCN electrolyte at the cell level, we compared the long‐term cycling stability of a Li‐S cell for the three electrolytes at three conditions that differ in sulfur loading, C‐rate, and E/S ratio. First, we compared the three electrolytes at a discharging/charging rate of 0.2 C / 0.1 C (1.0 / 0.5 mA cm⁻^2^) and at a low E/S ratio (5 µl mg⁻^1^) with a high‐sulfur‐loaded cathode (3 mg_s_ cm⁻^2^). The LiSCN electrolyte showed a large discharge capacity (1365.8 mAh g⁻^1^) that is even larger than that of the LiBr electrolyte (1264.5 mAh g⁻^1^) (**Figure** **4**a). This is in sharp contrast with the low sulfur utilization (53 %) for the LiTFSI electrolyte caused by sulfur electrode passivation. Figure 4b compares the voltage profiles at the 21st cycle for the three electrolytes. The LiBr electrolyte exhibited large discharge capacities in the initial cycles; however, its operation stopped at the 21st cycle due to short‐circuit, as indicated by the voltage drop upon overcharging. Dendritic Li growth and excessive electrolyte decomposition on the Li surface may have caused this sudden death.^[^ ^40^ ^]^ For the LiTFSI electrolyte, discharge capacity was gradually decreased from 50 cycles due to the shortening of the lower voltage plateau (Figure S11, Supporting Information). This indicates that the electrode passivation problem became aggravated with cycling. In addition to the much larger discharge capacity, the LiSCN electrolyte exhibited much higher cycling stability than the other electrolytes, demonstrating the dual effects of SCN^−^ (suppressed sulfur electrode passivation and enhanced Li metal stability) at the cell level. When compared to the recently reported other electrolyte research using five different parameters (E/S ratio, C rate, sulfur loading, discharge capacity, and cycle number), our Li‐S coin cell level shows outstanding discharge capacity and capacity retention under the low E/S ratio and high C rate conditions (Figure 4c).

We investigated the cycling behavior of the cells at a higher E/S ratio of 8 µl mg⁻^1^ and higher C rate condition (0.3 C / 0.2 C) (Figure 4d). When increasing E/S up to 8, the cycling stability was enhanced by three times for the LiBr electrolyte because the problems caused by poor Li reversibility were mitigated. However, the sulfur electrode passivation intensified at the high C rate condition, resulting in 32% reduction of discharge capacity compared with the 0.2 C / 0.1 C operation condition. Similarly, the discharge capacities for the LiTFSI electrolyte were merely 65 % of those at the 0.2 C / 0.1 C condition. By contrast, for the LiSCN electrolyte, the discharge capacity at the 0.3 C / 0.2 C condition was ≈80% of those at the 0.2 C / 0.1 C condition. The smaller reduction in discharge capacity at the higher C rate condition corroborates the efficacy of the LiSCN electrolyte in addressing the electrode passivation problem. The longest cycling stability for the LiSCN electrolyte at the condition also confirms the Li metal stabilization effect.

We attempted to operate a Li‐S battery at a highly challenging condition of 3.8 mg_s_ cm⁻^2^ and a 0.4 C / 0.2 C rate. At this condition, sulfur utilization is significantly limited by electrode passivation unless an appropriate catalyst is incorporated into the sulfur cathode. Figure 4e shows the cycling stability of the Li‐S cells at this challenging condition for the three. Similar to the results in the previous conditions, the LiSCN electrolyte showed outstanding performance in terms of discharge capacity and cycling stability. The voltage plateau of the LiTFSI electrolyte did not exhibit a lower voltage plateau as seen in the second cycle voltage profiles (Figure S12, Supporting Information) due to significant electrode passivation at a high discharge rate (2.54 mA cm⁻^2^). Conversely, the LiBr and LiSCN electrolytes revealed a lower voltage plateau with a lower to upper plateau ratio of 2.11 and 2.40, respectively, owing to the solution‐mediated 3‐D Li_2_S deposition. The operation of the LiBr electrolyte cell failed at 67 cycles due to short circuiting (comparatively, the LiSCN electrolyte failed at 100 cycles).

To demonstrate the possibility of the LiSCN electrolyte in developing a pouch‐type battery, a 6‐cell stack pouch design was fabricated using an LiSCN electrolyte. To improve the energy density of the Li‐S pouch cell, a lean electrolyte (E/S = 4 µl mg⁻^1^) condition was adopted and a 40 µm thin Li metal electrode was used for an N/P ratio of 2.35. The structure and optical image of our pouch cell are provided in Figure S13 (Supporting Information). The Li‐S pouch delivers an energy density of 313.6 Wh kg⁻^1^ and can be stably operated for 45 cycles (Figure 4f). The weight fraction of the Li‐S pouch cell is provided in Table S2 (Supporting Information). As shown in the voltage profile provided in Figure S14 (Supporting Information), the upper to lower plateau capacity ratio for the pouch cell is 2.74, which indicates the suppression of the sulfur electrode passivation in the pouch cell. These remarkable full‐cell performances suggest the possibility of realizing a high‐performance Li‐S battery by improving Li metal stability and suppressing electrode passivation through the electrolyte anion design.

## 3 Conclusion

In this paper, we demonstrated a new type of lithium salt anion, SCN^−^, which can improve sulfur utilization and Li metal stability. We unraveled the electron‐donating and electron‐accepting properties of SCN^−^ in the electrolyte by combining spectroscopic analysis and MD simulations. Due to the strong Li^+^ coordination by SCN^−^ and direct interaction of SCN^−^ with PSs, the LiSCN electrolyte demonstrated a remarkably high LiPS solubility, leading to intensified solution‐mediated 3‐D Li_2_S morphology. We identified that the SCN^−^‐coordinated Li^+^ solvation structure facilitates the formation of the Li_3_N‐rich SEI layer, which improves the reversibility of the Li metal electrode. For various cathode sulfur loadings and operating conditions, the dual effect of SCN^−^ was consistently observed at the cell level. Furthermore, the efficacy of the LiSCN electrolyte was confirmed for a Li‐S pouch cell. Our work suggests that a new solvation chemistry derived from a Janus type anion can address cathode passivation and the unstable Li/electrolyte interphase problem.

## 4 Experimental Section

### Electrolyte Characterization

Prior to making an electrolyte, lithium thiocyanate hydrate (LiSCN · xH_2_O, Sigma‐Aldrich) was fully dried in a vacuum oven at 100 ^o^C for 24 h and kept in an argon filled glove box. A base electrolyte solution was prepared in the argon filled glove box by dissolving 0.2 m lithium nitrate (LiNO_3_, Sigma‐Aldrich) and 0.2 m lithium bis(trifluoromethanesulfonyl)imide (LiTFSI, 3 m) in a 1:1 mixture of 1,3‐dioxolane (DOL, Sigma‐Aldrich) and 1,2‐dimethoxyethane (DME, Sigma‐Aldrich). Then, 0.8 m LiSCN, LiTFSI, and lithium bromide (LiBr, Sigma‐Aldrich) in the base electrolyte were individually prepared, which are denoted as LiSCN, LiTFSI, and LiBr electrolyte, respectively. 0.2 m Li_2_S_8_ catholytes were prepared by mixing and heating the electrolytes containing stoichiometric amounts of lithium sulfide (Li_2_S, Sigma‐Aldrich) and octa‐sulfur (S_8_, Sigma‐Aldrich) powder, and used as a cathode active material. To analyze the relative PS solubility for the electrolytes, UV–vis absorption spectroscopy (GENESYS 10S, Thermo Scientific) was used. For the UV–vis analysis, 1.6 m Li_2_S_4_ was prepared in the LiTFSI, LiBr, and LiSCN electrolytes. The supernatant of each catholyte was diluted by 500‐fold and analyzed using a 10 mm high precision cell (Hellma Analytics).^13^C Nuclear magnetic resonance (NMR) spectroscopy measurements were carried out on an Agilent 400 MHz 54 mm NMR DD2 (Agilent Technology) with 0.05 m tetramethylsilane (TMS, Sigma‐Aldrich) in dimethyl sulfoxide (DMSO‐d_6_, Sigma‐Aldrich) as a reference solution. Electrolyte samples for the NMR analysis were prepared by mixing 650 µl of catholyte or electrolyte and 100 µl of DMSO‐d_6_. The interaction between the polysulfide and SCN^−^ anion was evaluated using a dispersive Raman spectrometer (ARAMIS, JY Horiba) with a 514 nm wavelength laser.

### Sulfur Electrode Preparation and Characterization

A carbon nanofiber (CNF) electrode was fabricated by the electrospinning method and used as a positive electrode of a Li‐S cell. First, 1 g of poly acrylonitrile (PAN, Mw = 150 000, Sigma‐Aldrich) was dissolved in 10 mL of N,N‐dimethylformamide (DMF, Sigma‐Aldrich). The mixture was then stirred for 12 h. This uniform polymer solution was filled in a syringe (21 gauge) and a voltage of 17 kV was applied between the syringe and an aluminum foil collector to conduct electrospinning at a feeding rate of 1 mL h⁻^1^. The electro‐spun membrane was detached from the foil, dried overnight in a 60 °C oven, and punched into 18 pi disk size. The membrane was stabilized in an ambient air condition at 220 °C for 3 h, and then carbonized at 800 °C under an N_2_ flow condition (300 square cc min⁻^1^) for 1 h. The resulting CNF electrode was characterized using SEM and EDAX (Figure S1, Supporting Information). The pristine CNF electrode did not include solid state sulfur. Instead, 21 µl of 0.2 m Li_2_S_8_ solution was impregnated in DME into the electrode and the DME solvent was evaporated at 90 °C. The specific capacity of the cells was calculated based on the areal loading of Li_2_S_8_. The electrode morphologies were observed by using a scanning electron microscope (SEM, Magellan 400, FEI Co.). For the long‐term cycling test, a high sulfur‐loaded electrode (3.8 mg_s_ cm⁻^2^) provided by LG Energy Solution Ltd. was used. X‐ray photoelectron spectroscopy (XPS, K‐alpha, Thermo VG Scientific) was used to investigate the component of the SEI layer formed on Li metal.

### Electrochemical Analysis

For electrochemical analysis, a 2032‐type coin cell was used. A galvanostatic charge and discharge test was performed with a WBC300L (Wonatech) automatic battery cycler. For the Li symmetric cell test, two Li foils (150 µm, Honjo Metal) with a size of Ø 12 mm and Ø 16 mm were installed in the coin cell. For effective pressurization, the 40 µm‐thick Li metal foil was supported by an 1T SUS spacer in the Li/Li symmetric cells. A Celgard 2400 monolayer polypropylene (PP) membrane (Ø 18 mm) was inserted between the Li electrodes as a separator, and a predetermined amount of electrolytes (20 µl) was added to the cell. The Li symmetric cells were operated in a 1 mA cm⁻^2^ / 1 mA h cm⁻^2^ condition for testing the reversibility of the Li metal electrode. Chronoamperometry (CA) analysis was performed with a coin cell consisting of a CNF (Ø 12 mm) positive electrode, Li foil (Ø 16.5 mm, 450 µm) negative electrode, PP separator (Ø 18 mm), and catholyte. The cell voltage was first kept at 2.2 V to form the short chain PSs and decreased to 2.05 V stepwise to conduct the nucleation and growth of Li_2_S. Before the galvanostatic cycling test, Li‐S cells were subjected to pre‐cycling at 0.05 C for 2 cycles.

### Computer Simulation

All density functional theory (DFT) calculations were performed using the DMol3 package in Material Studio software. The generalized gradient approximation (GGA) method, Perdew‐Burke‐Ernzerhof (PBE) functional, and double numerical plus polarization (DNP+) basis set were used to optimize the geometries and calculate the binding energies. The orbital cutoff was set as 4.0 Å and the COSMO model was applied to reflect the implicit DME solvent conditions. Atomistic molecular dynamics (MD) simulations were performed using the Forcite package in Material Studio software. 0.8 m LiX (X=SCN, Br, TFSI) was imitated in the base electrolyte by constructing a simulation cell including 241 DME, 357 DOL, 60 Li^+^, 40 X^−^ (X^−^ = SCN^−^, Br^−^, TFSI^−^), 10 TFSI^−^, and 10 NO_3_^−^ molecules. The COMPASS III force field, provided by Material Studio software, was assigned to all molecules. To investigate the coordination of Li^+^, the constructed cells were equilibrated at a 1 atm, 298 K condition in an NPT ensemble during 1 ns. A Nose thermostat and Berndensen barostat with an integration time step of 1 fs was used to equilibrate the simulation cells under constant temperature and pressure. 1 ns of NVT ensemble simulation was followed to equilibrate the cells at the same temperature. In the equilibrium condition, we ran the simulation for 5 ns and exhibited the coordination chemistry among the molecules through the radial distribution function (RDF).

## Conflict of Interest

The authors declare no conflict of interest.

## Supporting information

Supporting Information

## Data Availability

The data that support the findings of this study are available on request from the corresponding author. The data are not publicly available due to privacy or ethical restrictions.

## References

[1] S.‐E. Cheon , K.‐S. Ko , J.‐H. Cho , S.‐W. Kim , E.‐Y. Chin , H.‐T. Kim , J. Electrochem. Soc. 2003, 150, A800.

[2] D.‐R. Chang , S.‐H. Lee , S.‐W. Kim , H.‐T. Kim , J. Power Sources 2002, 112, 452.

[3] S.‐E. Cheon , K.‐S. Ko , J.‐H. Cho , S.‐W. Kim , E.‐Y. Chin , H.‐T. Kim , J. Electrochem. Soc. 2003, 150, A796.

[4] Q. Jin , X. Qi , F. Yang , R. Jiang , Y. Xie , L. Qie , Y. Huang , Energy Storage Mater. 2021, 38, 255.

[5] M. Zhao , B.‐Q. Li , H.‐J. Peng , H. Yuan , J.‐Y. Wei , J.‐Q. Huang , Angew. Chem., Int. Ed. 2020, 59, 12636. 10.1002/anie.201909339 31490599

[6] Y. X. Ren , T. S. Zhao , M. Liu , P. Tan , Y. K. Zeng , J. Power Sources 2016, 336, 115.

[7] Y.‐C. Chien , A. S. Menon , W. R. Brant , M. J. Lacey , D. Brandell , J. Phys. Chem. C 2022, 126, 2971.

[8] H. Pan , K. S. Han , M. H. Engelhard , R. Cao , J. Chen , J.‐G. Zhang , K. T. Mueller , Y. Shao , J. Liu , Adv. Funct. Mater. 2018, 28, 1707234.

[9] Z. Li , Y. Zhou , Y. Wang , Y.‐C. Lu , Adv. Energy Mater. 2019, 9, 1802207.

[10] A. Gupta , A. Bhargav , A. Manthiram , Adv. Energy Mater. 2019, 9, 1803096. 31807123 10.1002/aenm.201803096 PMC6894182

[11] H. Chu , J. Jung , H. Noh , S. Yuk , J. Lee , J.‐H. Lee , J. Baek , Y. Roh , H. Kwon , D. Choi , K. Sohn , Y. Kim , H.‐T. Kim , Adv. Energy Mater. 2020, 10, 2000493.

[12] H. Chu , H. Noh , Y.‐J. Kim , S. Yuk , J.‐H. Lee , J. Lee , H. Kwack , Y. Kim , D.‐K. Yang , H.‐T. Kim , Nat. Commun. 2019, 10, 188. 30643115 10.1038/s41467-018-07975-4 PMC6331553

[13] M. Baek , H. Shin , K. Char , J. W. Choi , Adv. Mater. 2020, 32, 2005022. 10.1002/adma.202005022 33184954

[14] A. Gupta , A. Bhargav , A. Manthiram , ACS Energy Lett. 2021, 6, 224. 34212110 10.1021/acsenergylett.0c02461 PMC8243416

[15] H. Pan , X. Wei , W. A. Henderson , Y. Shao , J. Chen , P. Bhattacharya , J. Xiao , J. Liu , Adv. Energy Mater. 2015, 5, 1500113.

[16] A. Elabd , J. Kim , D. Sethio , S. Kang , T. Kang , J. W. Choi , A. Coskun , ACS Energy Lett. 2022, 7, 2459.

[17] N. B. Aetukuri , B. D. McCloskey , J. M. Garcia , L. E. Krupp , V. Viswanathan , A. C. Luntz , Nat. Chem. 2015, 7, 50. 25515890 10.1038/nchem.2132

[18] L. Johnson , C. Li , Z. Liu , Y. Chen , S. A. Freunberger , P. C. Ashok , B. B. Praveen , K. Dholakia , J.‐M. Tarascon , P. G. Bruce , Nat. Chem. 2014, 6, 1091. 25411888 10.1038/nchem.2101

[19] C. Wechwithayakhlung , D. M. Packwood , D. J. Harding , P. Pattanasattayavong , J. Phys. Chem. Solids 2021, 154, 110085.

[20] P. Cauliez , V. Polo , T. Roisnel , R. Llusar , M. Fourmigué , CrystEngComm 2010, 12, 558.

[21] M. Schmeisser , P. Illner , R. Puchta , A. Zahl , R. van Eldik , Chemistry 2012, 18, 10969. 22806990 10.1002/chem.201200584

[22] G. Hu , D. Han , G. Jia , T. Chen , Z. Feng , C. Li , J. Raman Spectrosc. 2009, 40, 387.

[23] Q. Zou , Y.‐C. Lu , J. Phys. Chem. Lett. 2016, 7, 1518. 27050386 10.1021/acs.jpclett.6b00228

[24] N. Zhong , C. Lei , R. Meng , J. Li , X. He , X. Liang , Small 2022, 18, 2200046. 10.1002/smll.202200046 35266288

[25] M. Cuisinier , C. Hart , M. Balasubramanian , A. Garsuch , L. F. Nazar , Adv. Energy Mater. 2015, 5, 1401801.

[26] B. Yang , H. Jiang , Y. Zhou , Z. Liang , T. Zhao , Y.‐C. Lu , ACS Appl. Mater. Interfaces 2019, 11, 25940. 31246006 10.1021/acsami.9b07048

[27] Q. He , Y. Gorlin , M. U. M. Patel , H. A. Gasteiger , Y.‐C. Lu , J. Electrochem. Soc. 2018, 165, A4027.

[28] M. M. Tylka , J. L. Willit , M. A. Williamson , J. Electrochem. Soc. 2017, 164, H5327.

[29] M. Jafarian , M. G. Mahjani , F. Gobal , I. Danaee , J. Electroanal. Chem. 2006, 588, 190.

[30] M. A. Lowe , J. Gao , H. D. Abruña , RSC Adv. 2014, 4, 18347.

[31] Y.‐C. Lu , Q. He , H. A. Gasteiger , J. Phys. Chem. C 2014, 118, 5733.

[32] J.‐T. Yeon , J.‐Y. Jang , J.‐G. Han , J. Cho , K. T. Lee , N.‐S. Choi , J. Electrochem. Soc. 2012, 159, A1308.

[33] J. D. McBrayer , T. E. Beechem , B. R. Perdue , C. A. Apblett , F. H. Garzon , J. Electrochem. Soc. 2018, 165, A876.

[34] H.‐K. Kang , S.‐G. Woo , J.‐H. Kim , S.‐R. Lee , Y.‐J. Kim , Electrochim. Acta 2015, 176, 172.

[35] C. Liu , T. Li , H. Zhang , Z. Song , C. Qu , G. Hou , H. Zhang , C. Ni , X. Li , Sci. Bull. 2020, 65, 434. 10.1016/j.scib.2019.11.014 36747432

[36] X.‐B. Cheng , C. Yan , X. Chen , C. Guan , J.‐Q. Huang , H.‐J. Peng , R. Zhang , S.‐T. Yang , Q. Zhang , Chem 2017, 2, 258.

[37] A. Majumdar , S. C. Das , T. Shripathi , R. Hippler , Compos. Interfaces 2012, 19, 161.

[38] Y.‐J. Kim , S. H. Kwon , H. Noh , S. Yuk , H. Lee , H. s. Jin , J. Lee , J.‐G. Zhang , S. G. Lee , H. Guim , H.‐T. Kim , Energy Storage Mater. 2019, 19, 154.

[39] Z. Liu , Y. Wang , Int. J. Electrochem. Sci. 2016, 11, 1434.

[40] L. Shi , C. S. Anderson , L. Mishra , H. Qiao , N. Canfield , Y. Xu , C. Wang , T. Jang , Z. Yu , S. Feng , P. M. Le , V. R. Subramanian , C. Wang , J. Liu , J. Xiao , D. Lu , Adv. Sci. 2022, 8, 2201640. 10.1002/advs.202201640 PMC9313511 35524632

[41] L.‐P. Hou , X.‐Q. Zhang , N. Yao , X. Chen , B.‐Q. Li , P. Shi , C.‐B. Jin , J.‐Q. Huang , Q. Zhang , Chem 2022, 8, 1083.

[42] Y. Q. Peng , M. Zhao , Z. X. Chen , Q. Cheng , Y. Liu , C. X. Zhao , X. Ma , B. Q. Li , C. M. Chen , J. Q. Huang , Q. Zhang , Batter Supercaps 2021, 5, e202100359.

