# Supplementary material for: Confronting Sulfur Electrode Passivation and Li Metal Electrode Degradation in Lithium‐Sulfur Batteries Using Thiocyanate Anion
Source: Adv Sci (Weinh). 2023 Mar 21;10(15):2301006. doi: 10.1002/advs.202301006 (PMC10214224; doi:10.1002/advs.202301006)
Supplement: Supplementary file 1 — Supporting Information [file ADVS-10-2301006-s001.pdf]

ADVANCED SCIENCE
Open Access

Supporting Information

for *Adv. Sci.*, DOI 10.1002/advs.202301006

Confronting Sulfur Electrode Passivation and Li Metal Electrode Degradation in Lithium-Sulfur Batteries Using Thiocyanate Anion

*Jinkwan Jung, Hyunwon Chu, Ilju Kim, Dong Hyun Lee, Gisu Doo, Hyeokjin Kwon, Wonhee Jo, Sejin Kim, Hyenah Cho and Hee-Tak Kim**

Supplementary Information

Confronting sulfur electrode passivation and Li metal electrode degradation in lithium sulfur batteries using thiocyanate anion

Jinkwan Jung [a], Hyunwon Chu [a], Il Ju Kim [a], Dong Hyun Lee [a], Gisu Doo [a], Hyeokjin Kwon [a], Wonhee Jo [a], Sejin Kim [a], Hyenah Cho [a], and Hee-Tak Kim [*a,b]

[a]*Department of Chemical and Biomolecular Engineering, Korea Advanced Institute of Science and Technology, 291 Daehak-ro, Yuseong-gu, Daejeon 34141, Republic of Korea*

[b]*Advanced Battery Center, KAIST Institute for the NanoCentury, Korea Advanced Institute of Science and Technology, 291 Daehak-ro, Yuseong-gu, Daejeon 34141, Republic of Korea*

* Corresponding author: Tel.: +82-42-350-3916; Fax: +82-42-350-3910

E-mail: heetak.kim@kaist.ac.kr (H. –T. Kim)

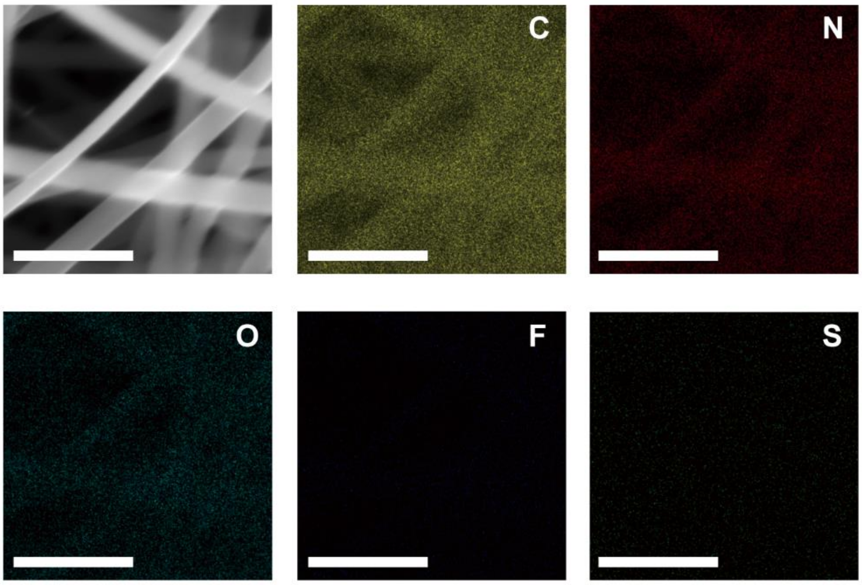

Figure S 1. SEM image and element mapping images of the pristine carbon nanofiber (CNF) electrode. The elemental mapping of C (yellow), N (red), O (sky blue), F (blue), S (green) were conducted using scanning electron microscopy-energy dispersive X-ray spectroscopy (SEM-EDS). (Scale bars: 1 μm)

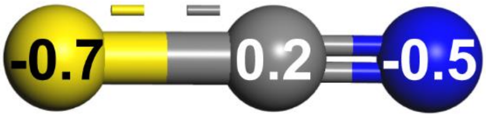

Figure S 2. Partial charges of the SCN anion calculated by the Mulliken charge analysis method of DFT calculations.

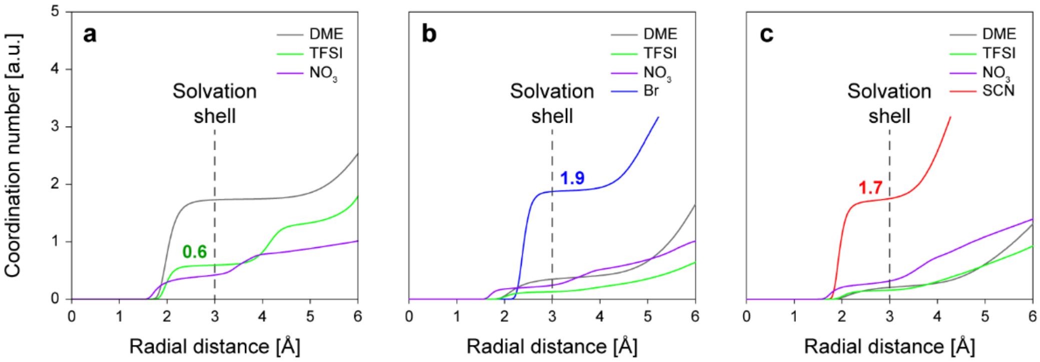

Figure S 3. Coordination number of Li^+ in the three different electrolytes. Coordination number (N(r)) of Li^+ for the anion and solvent molecules in a) the LiTFSI, b) LiBr, and c) LiSCN electrolytes. Dotted lines indicate the criterion of the first solvation shell. The equation used for the calculation of type i molecule coordination number, $N_i(r)$, is given as

follows.

$$N_i(r) = 4\pi n_j \int_0^{R_M} g_{ij}(r) r^2 dr$$

In this equation, $g_{ij}(r)$ is a radial distribution function between i and j type molecules with distance, R_M is the first solvation shell criterion with a distance of the first minimum of $g_{ij}(r)$, and n_j is the number density of the j type molecules. A molecule with high electron donicity tends to coordinate more with Li^+, which is indicated by a high coordination number.

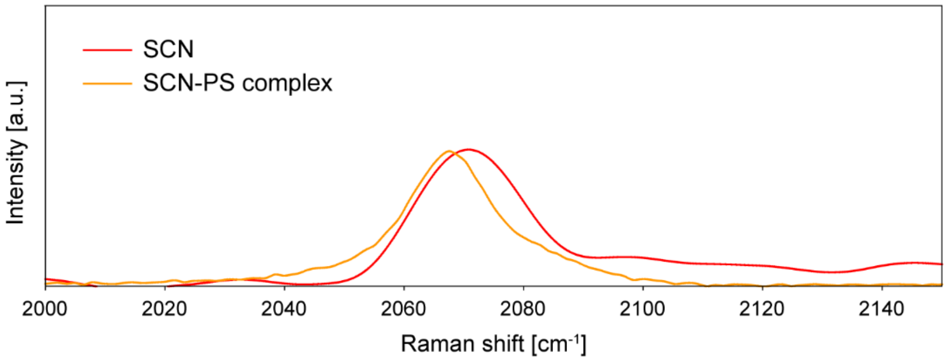

Figure S 4. Raman spectra of the pure and 1.6 M Li_2S_4 added LiSCN electrolytes.

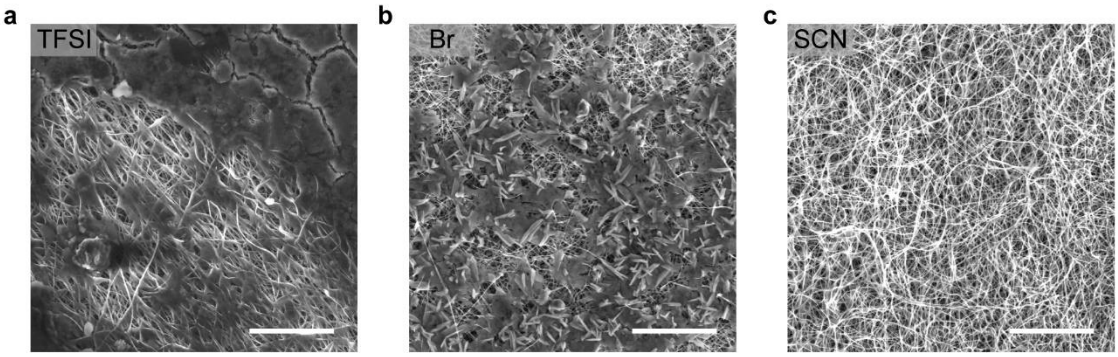

Figure S 5. Large scale SEM images of the Li_2S deposition morphologies for a) the LiTFSI, b) LiBr, and c) LiSCN electrolytes after a discharge at 0.4 C. (Scale bars: 20 μm).

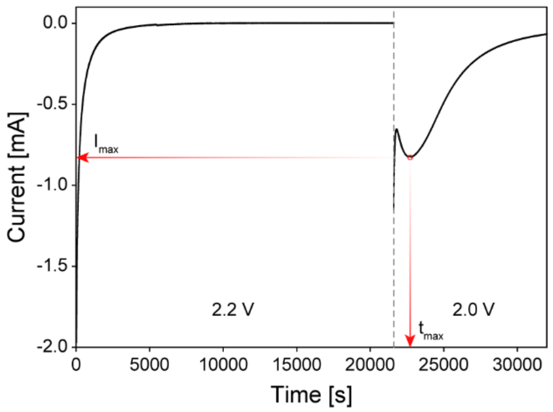

Figure S 6. The typical current profile with time during Chronoamperometry analysis.

Chronoamperometry analysis to investigate the Li_2S nucleation and growth. Prior to Li_2S nucleation and growth, we applied 2.2 V for 6 h to Li-S cells to convert long chain polysulfides to short chain polysulfides. Then, Li_2S nucleation and the growth mode were investigated at 2.0 V. I_{max} and the t_{max} are identified in this figure. The following equations were used to fit the model to the data.

$$2D-progressive\ (2DP):\ \frac{I}{I_{max}}=\left(\frac{t}{t_{max}}\right)^2 exp\left[-\frac{2}{3}\left(\frac{t^3-t_{max}^3}{t_{max}^3}\right)\right] \qquad (S1)$$

$$2D-instantaneous\ (2DI):\ \frac{I}{I_{max}}=\left(\frac{t}{t_m}\right) exp\left[-\frac{1}{2}\left(\frac{t^2-t_{max}^2}{t_{max}^2}\right)\right] \qquad (S2)$$

$$3D-progressive\ (3DP):\ \left(\frac{I}{I_{max}}\right)^2=\left(\frac{1.2254}{t/t_{max}}\right)\left[1-exp\left(-2.3367\frac{t^2}{t_{max}^2}\right)\right]^2 \qquad (S3)$$

$$3D-instantaneous\ (3DI):\ \left(\frac{I}{I_{max}}\right)^2=\left(\frac{1.9542}{t/t_{max}}\right)\left[1-exp\left(-1.2564\frac{t}{t_{max}}\right)\right]^2 \qquad (S4)$$

	TFSI	Br	SCN
t_m [s]	228.31	644.58	734.48
I_m [mA]	0.80	2.50	2.58
$N_0k_g^2$ [s^{-2}]	1.44*10^{-10}	5.06*10^{-11}	3.81*10^{-11}
N_0	282175	950158	646308

Table S1. List of t_m, I_m, $N_0k_g^2$, and N_0 values determined from the CA analysis for the LiTFSI, LiBr, an LiSCN electrolytes.

For the quantification of lateral growth rate ($N_0k_g^2$) and nuclei density (N_0), the equations of S5 to S8 were used. In these equations, F is the Faraday constant, A is the surface area of the working electrode, M is the molar weight of the Li_2S, $\boldsymbol{\rho}$ is the deposit density, D_i is the diffusion coefficient of the electroactive species, and C_i is the concentration of the electroactive species.

$$N_0 = \frac{I_m^2}{0.41n^2F^2D_i^2C_i^2A^2k_i} \qquad \text{(S5)}$$

$$k_i = \left(\frac{8\pi C_i M}{\rho}\right)^{1/2} \qquad \text{(S6)}$$

$$t_m = \left(\frac{\rho^2}{2\pi N_0 M^2 k_g^2}\right)^{1/2} \qquad \text{(S7)}$$

$$I_m = nFhk_g(2\pi N_0)^{1/2}\exp\left(-\frac{1}{2}\right) \qquad \text{(S8)}$$

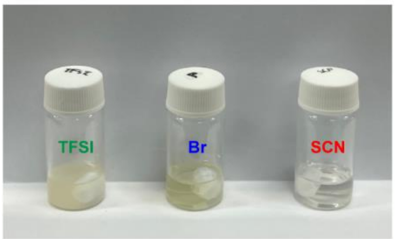

Figure S 7. Li_2S solubility test for three different electrolytes (TFSI, Br, and SCN electrolytes). 4 mM Li_2S in LiTFSI electrolyte, 8 mM Li_2S in LiBr electrolyte, and 10 mM Li_2S in LiSCN electrolyte, respectively.

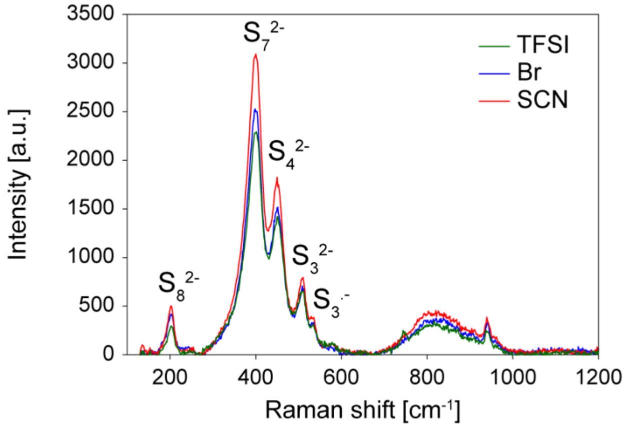

Figure S 8. Raman spectroscopy spectra of the 1.6 M Li_2S_4 containing LiTFSI, LiBr, and LiSCN electrolytes.

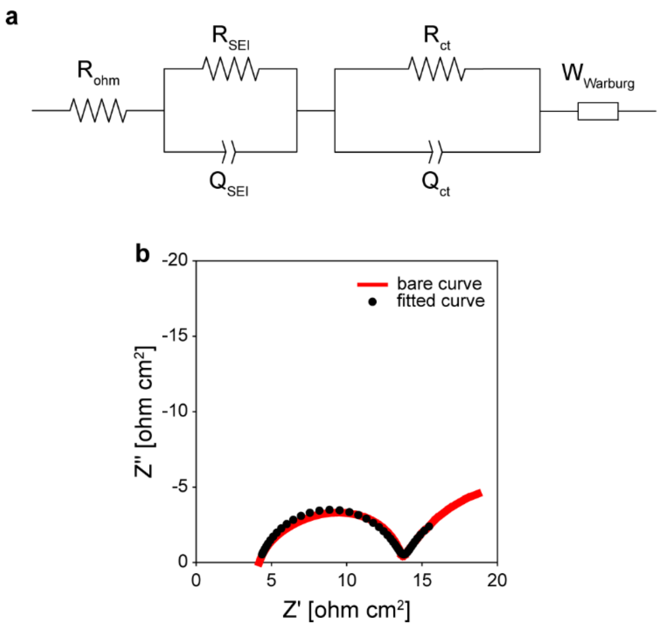

Figure S 9. a) An equivalent circuit model of the operated Li/Li symmetric cell. b) Typical Nyquist plot of the Li/Li symmetric cell with a LiSCN electrolyte operated for 30 cycles. The circuit model fitting result is compared. The interfacial resistance is the sum of the charge transfer resistance (R_{ct}) and SEI resistance (R_{SEI}).

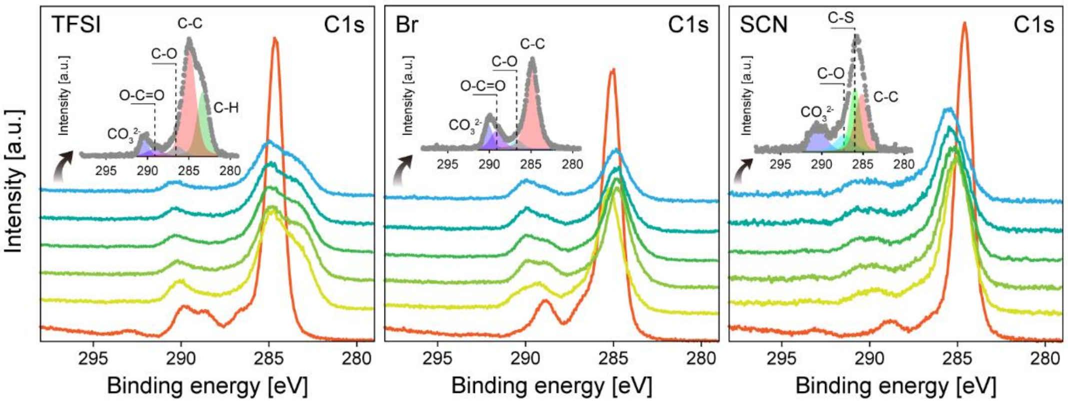

Figure S 10. XPS C1s spectra of the Li metal anodes taken out of the Li symmetric cells with the LiTFSI, LiBr, and LiSCN electrolytes after 20 cycles.

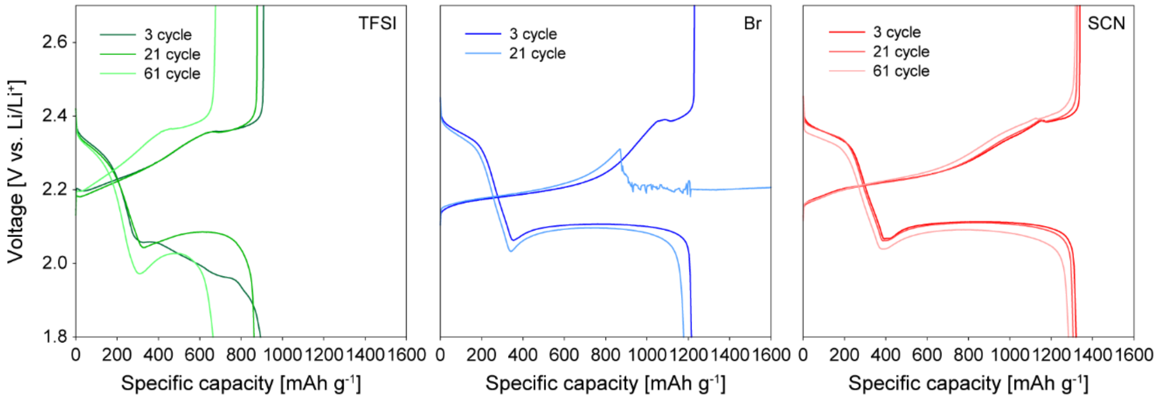

Figure S 11. The discharge-charge voltage profiles of the Li-S cells with the LiTFSI, LiBr and LiSCN electrolyte at 3, 21, and 61 cycles. The E/S ratio was 5 µl mg^{-1}, and discharging/charging rate was 0.2 C/0.1 C.

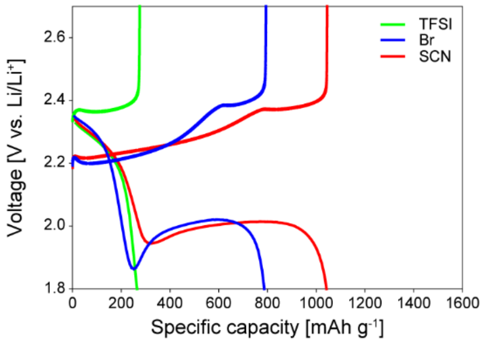

Figure S 12. Voltage profiles at second cycle of Li-S cells at an E/S = 10 µl mg^{-1}, 3.8 mg$_s$ cm^{-2}, and 0.4 C / 0.2 C rate for the three electrolytes (LiTFSI, LiBr, LiSCN electrolytes).

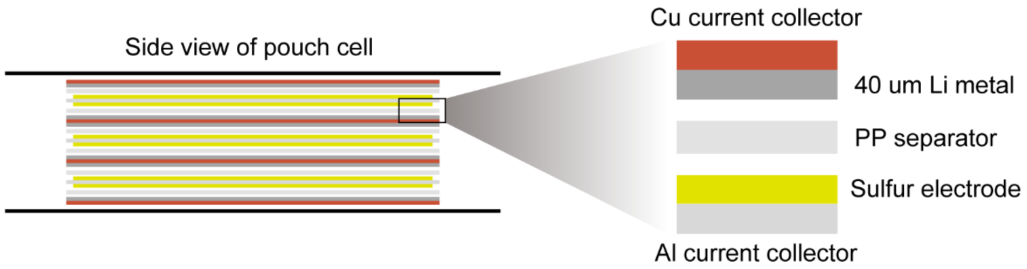

Figure S 13. Scheme of the Li-S pouch cell. A 3.2 mg_s cm^{-2} loaded sulfur electrode provided by LG Energy Solution was used for the pouch cell.

	Weight of pouch cell components [g]
Cathode	0.288
Li foil	0.192
Separator	0.166
Cu foil	0.645
Al foil	0.316
Electrolyte	1.070

Table S 2. Parameters of Li-S pouch cell.

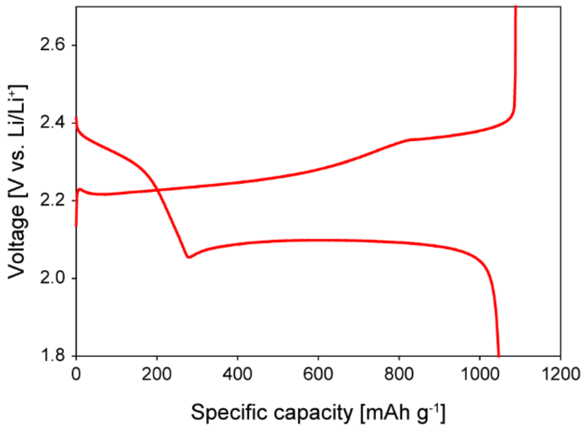

Figure S 14. First cycle voltage profile of the Li-S pouch cell with the LiSCN electrolyte.
